# Gross Changes in Adrenal Glands in Suicidal and Sudden Death Cases: A Postmortem Study

**DOI:** 10.7759/cureus.51175

**Published:** 2023-12-27

**Authors:** Sangita Chaurasia, Ruchi Ganvir, Rajneesh K Pandey, Saagar Singh, Jayanthi Yadav, Reeni Malik, Sneha Choubal, Arneet Arora

**Affiliations:** 1 Department of Forensic Medicine and Toxicology, Gandhi Medical College, Bhopal, IND; 2 Department of Microbiology, Atal Bihari Vajpayee Government Medical College, Vidisha, IND; 3 Department of Forensic Medicine and Toxicology, Shyam Shah Medical College, Rewa, IND; 4 Department of Forensic Medicine and Toxicology, All India Institute of Medical Sciences, Bhopal, IND; 5 Department of Pathology, Gandhi Medical College, Bhopal, IND; 6 Department of Pathology, Parakh Pathology Laboratory, Bhopal, IND

**Keywords:** chronic stress, acute stress, suicide, gross changes, adrenal gland

## Abstract

Introduction

Chronic stress breaches the normal homeostasis of the hypothalamic-pituitary-adrenal axis, leads to chronic adrenal fatigue, and causes hypertrophy and hyperplasia of the adrenal gland. The current study was carried out with the aim of observing the difference in gross morphological changes in the adrenals of people dying by suicide and from sudden death, as persons committing suicide are exposed to chronic stress (depression), while those dying suddenly are exposed to the acute stress of dying.

Materials and methods

The present analytical study was carried out in the Department of Forensic Medicine and Toxicology, Gandhi Medical College, Bhopal, India, after approval from the Institutional Ethics Committee (IEC). A total of 100 established cases of suicide with prominent autopsy findings and relevant history without signs of decomposition, aged 15-60 years, irrespective of gender, and a variable survival period (immediate death to within 24 hours) were selected. A total of 20 controls included those who died suddenly from an act other than suicide within 24 hours of the incident. Due consent was obtained from the relatives and police in the prescribed proforma. Kidneys, along with peri-renal fat and adrenal glands on both sides, were carefully removed and examined.

Results

A total of 25% of suicide victims reported a history of chronic stress, 13% self-destructive behavior, 3% untreated depression, and 8% reported financial or marital difficulties. The right adrenal gland was found to be heavier than the left in the control group. In addition, both left and right adrenal glands weighed more in males. Among the suicidal cases, the weight of the left adrenal gland was greater than that of the right, and the weight of the gland in males was higher than that in females. The difference in adrenal gland weight among males was significant in both case and control groups (combined p-value = 0.0001) but was insignificant in females, probably due to their disproportionate ratio in both groups. There was no significant relationship between adrenal gland weight and individual age or weight. However, adrenal gland weight in both groups was significantly associated with the height of the individual (p-value = 0.001 in the study group and < 0.05 in the control group). The difference in adrenal gland volume between the suicidal and control groups was not significant, indicating that the increase in size is not a differentiating criterion for acute and chronic stress. The relative adrenal gland weight was significantly higher in the suicidal group.

Conclusion

The external appearance of the adrenal gland may be regarded as a normal response to stress in relation to the mode of death. The left adrenal gland is more likely to show an increase in weight in response to chronic stress. The weight of the adrenal gland in both groups is significantly associated with the height of the deceased. Relative adrenal weight can be considered as specific for suicidal cases exposed to chronic stress. However, the volume of the adrenal gland may be considered an unreliable criterion in the differentiation of chronic stress from acute stress.

## Introduction

The WHO has defined suicide as deliberate harm to oneself and self-injury with varying degrees of lethal interest and a fatal outcome [1]. In 2008, the WHO claimed that Japan, China, and India might account for approximately 40% of the world's suicide cases. Moreover, 39% of suicides in lower-middle-income countries (LMICs) are reported in Southeast Asia [2]. The incidence of suicide in India in 2003 was 10.4%, increased to 11.0% in 2013, and came up to 14% as per the 2023 data [3]. The majority of those committing suicide suffer from some type of stress, depression, or other emotional disorder. Acute and ongoing stress can lead to suicidal behavior, which is associated with diagnosable psychiatric disorders [4]. The major stress hormone that the adrenal gland releases is the steroid hormone cortisol. The anterior pituitary gland releases adrenocorticotropic hormone into the bloodstream; this hormone travels to the adrenal cortex and stimulates the secretion of cortisol by endocrine cells. General adaptation syndrome, also known as hypothalamic-pituitary-adrenal axis activation, is responsible for this physiological adaptation to stress [5]. Chronic stress breaches the normal homeostasis of the hypothalamic-pituitary-adrenal axis, leads to chronic adrenal fatigue, and causes hypertrophy and hyperplasia of the adrenal gland. Several studies have suggested an association between hypothalamic-pituitary-adrenal axis dysregulation, stress, affective disorder, and suicidal behavior. Subsequently, the elevation of total plasma cortisol has been suggested as a prediction of suicide [6]. According to these studies, chronic stress-induced adrenal gland growth results in a change in adrenal function that may have an impact on suicidal behavior in depressed patients. The present study was performed to observe differences in gross morphological changes in the adrenal glands of people dying from suicide and from sudden death, as persons committing suicide are exposed to chronic stress (depression), while those dying suddenly are exposed to the acute stress of dying.

## Materials and methods

The present analytical and comparative observational study was performed in the Department of Forensic Medicine and Toxicology, Gandhi Medical College, Bhopal, India, after approval from the Institutional Ethics Committee with approval reference number 13419-20/MC/7/2015. One hundred established cases of suicide with prominent autopsy findings and relevant history without any signs of decomposition, ranging from 15 to 60 years of age, irrespective of gender, and with a variable survival period (immediate death to within 24 hours) were selected as study cases. Cases with doubtful or insufficient history, chronic gross pathology to any organ, hospitalization for >24 hours, alcohol ingestion at the time of autopsy, known cases of depression having taken or taking antidepressant treatment, ages <15 or >60 years, pregnant women, and mutilated bodies were excluded. Twenty control cases included those who had died suddenly by an incident other than suicide within 24 hours of that incident; such incidents included road traffic accidents, electrocution, acute cardiac cause without gross pathology, and homicidal death due to firearm, stabbing, or blunt-force injury. Due consent was obtained from relatives and police in the prescribed protocols. An electronic digital weighing scale with 20-gram accuracy measured the deceased's clothing-free weight. The height of the deceased was measured in the supine position using metallic ruler tape by placing one piece of cardboard at the head end and another piece at the foot end; the distance between these two cardboard pieces was measured. An external examination of the deceased body was performed. The internal examination included a meticulous examination of all visceral organs to exclude gross pathology in any organ.

Kidneys, along with peri-renal fat with adrenal glands on both sides, were removed carefully. Both kidneys were cleared of peri-renal fat and weighed individually using an electronic weighing machine (model no. IND/09/2001/28, Essae Teraoka Private Limited, Bengaluru, Karnataka, India) with an accuracy of 0.01 g. After removal, the kidneys and the adrenal glands were carefully separated (without tearing) from the peri-renal fat and examined grossly. Adrenal glands were dissected, and their outer surfaces were dried with blotting paper. Their sizes were measured using a metal ruler and weighed by means of the same digital electronic weighing machine, with an accuracy of 0.01 g.

**Figure 1:**
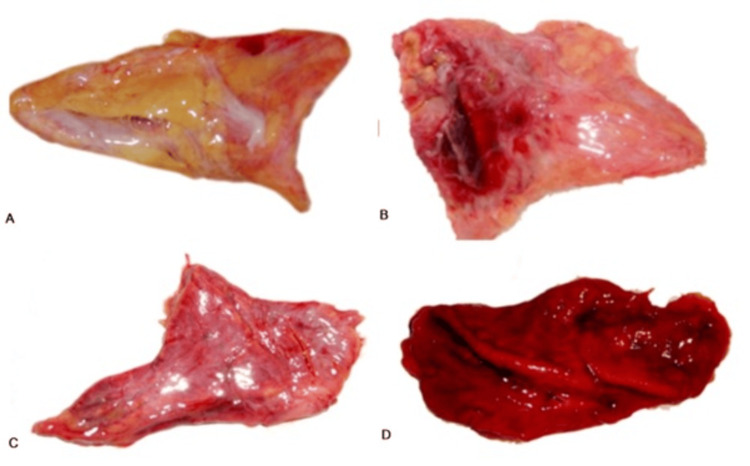
External appearance of adrenal gland A: Pale, B: Normal, C: Congested, D: Hemorrhagic

The procedure for examination of both adrenal glands in gross included the external appearance, size, and weight of the glands in comparison to the normal appearance (grade 2) of the gland. The external appearance is graded as Grade 1: Pale - The gland appeared yellowish-white on gross appearance with a normal contour. Grade 2: Normal - The gland being highly vascular showed the normal amount of blood vessels and normal contour. Grade 3: Congested - The gland appeared more reddish and shrunken in comparison to a normal gland. Grade 4: Hemorrhagic - The gland appeared profusely red (due to hemorrhage) and extremely shrunken (Figure 1).

## Results

**Figure 2:**
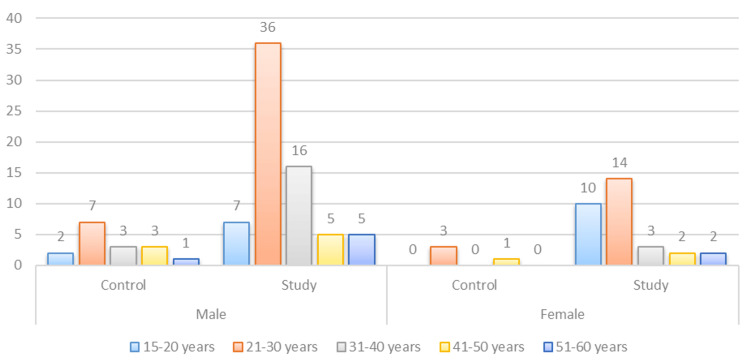
Distribution of cases and controls on the basis of age and gender

The present study included 100 suicide cases, representing 69 males and 31 females within the age range of 15 to 60 years, with a mean ± standard deviation age of 29.5 ± 11.4 years. Control cases included 16 males and four females, with a mean ± SD age of 33.3 ± 11.5 years (Figure 2).

**Figure 3:**
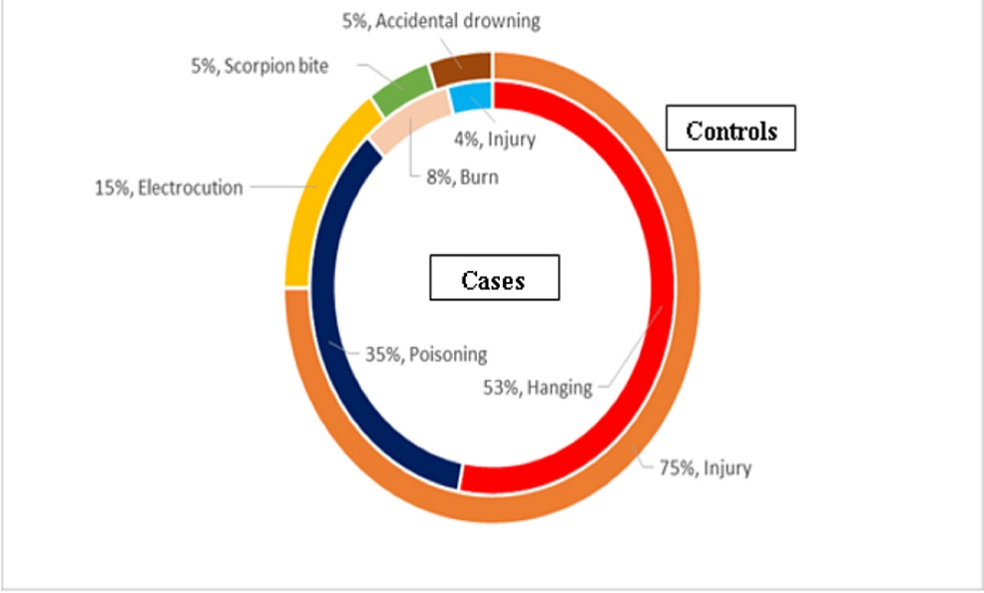
Distribution of cases and controls on the basis of cause of death

Of the suicidal cases, 60 belonged to low socioeconomic groups, 24 came from a moderate socioeconomic group, and 16 from higher socioeconomic status; 25 suicidal cases had a known history of psychiatric illness, out of which 13 subjects had exhibited self-destructive behavior in the form of recent self-inflicted wounds or older scars found on the body. A further three cases had a known history of depression without treatment, eight had a history of financial and marital stress, and one was suffering from postpartum psychosis. The methods of suicide used were hanging in 53% (n = 53), poisoning in 35% (n = 35), burning in 8% (n = 8), and other injuries in 4% (n = 4) (Figure 3).

Among the control group, 17 were from a low socioeconomic group and three were from a moderate-income group. Subjects with a known history of psychiatric illness or any form of financial, marital, or other stress were excluded from this group. Causes of death were injury (including head injury or multiple injuries due to a road traffic accident, homicidal stabbing, or homicidal throat-cutting injury) in 75% (n = 15), electrocution in 15% (n = 3), accidental drowning in 5% (n = 1), and scorpion bite in 5% (n = 1) (Figure 3).

The mean ± SD total body weight of individuals in the study group was 56.94 ± 8.39 kg and 62.6 ± 13.02 kg in the control group; the difference was statistically significant by unpaired t-test (p = 0.014, t = 2.48, df = 118). In the study group, the mean ± SD body weight of males and females was 59.3 ± 8.11 kg and 51.6 ± 6.53 kg, respectively, while the mean body weight in the control group was 66.31 ± 11.7 kg for males and 47.8 ± 4.20 kg for females. A statistically significant difference was found between the body weights of male individuals in the study and control groups (p = 0.05, t = 2.85, df = 83), but for females the difference was statistically insignificant (p = 0.27, t = 1.13, df = 33).

A statistically insignificant relationship was found between the mean ± SD heights of individuals in the study and control groups (unpaired t-test, p = 0.42, t = 0.80, df = 118) (Table 1).

**Table 1:** Comparison of gross morphological characteristics in study and control groups N: Number of subjects

Parameters	Groups				P-value
	Study group, N=100		Control group, N=20		
Weight (in kg) (average mean ±SD)					
Male	59.3±8.11		66.31±11.7		0.05*
Female	51.6±6.53		47.75±4.20		0.27
Total	56.94±8.39		62.6±13.02		0.014*
Height (in cm) (average mean ±SD)					
Male	170.1±7.16		172.1±5.38		0.29
Female	157.9±4.62		153.3±10.6		0.12
Total	166.3±8.54		168±9.83		0.424
Weight of kidney (in gms) (average mean ±SD)					
Right Kidney	113.4±11.33		117.9±12.82		0.114
Left Kidney	112.9±11.38		117.7±12.75		0.094
Volume of adrenals (in cm^3^) (average mean ±SD)					
Right	10.52±4.72		9.39±3.50		0.312
Left	9.46±4.35		8.44±4.17		0.337
Combined	19.98±8.29		17.83±6.89		0.275
Weight of adrenals (in gms) (average mean ±SD)					
Right	5.098±1.39		3.99±0.78		0.0009*
Left	5.28±1.45		3.84±0.9		<0.0001*
Combined	10.38±2.74		7.84±1.63		<0.0001*
Relative adrenal weight (gm/m^2^) (average mean ±SD)					
Right	1.85±0.50		1.42±0.24		0.0003*
Left	1.92±0.54		1.36±0.30		<0.0001*
Combined	3.76±1.01		2.78±0.53		0.0001*
Grade of the external appearance of adrenal glands, N (%)					
	100 cases	200 adrenals	20 cases	40 adrenals	-
Grade-1 (pale)	2 (2%)	4 (2%)	8 (40%)	14 (35%)	
Grade-2 (normal)	1 (1%)	2 (1%)	7 (35%)	12 (30%)	
Grade-3 (congested)	37 (37%)	7 (35%)	8 (40%)	14 (35%)	
Grade-4 (hemorrhagic)	60 (60%)	12 (30%)	0 (0%)	0 (0%)	

The difference in weight of the right and left kidneys among the suicidal and control groups was not statistically significant (p = 0.114, t = 1.59, df = 118 for the right kidney and p = 0.094, t = 1.68, df = 118 for the left kidney) (Table 1).

The volume of the adrenal gland (in cm^3^) was calculated by multiplying the total length, width, and height (thickness). The mean ± SD combined volume of both adrenal glands in the study group was 19.98 ± 8.20 cm^3^, which was not significantly higher than the mean ± SD combined volume of adrenal glands in the control group (unpaired t-test, p = 0.275, t = 1.09, df = 118). In the study group, the mean ± SD volume of the right adrenal gland was 10.52 ± 4.72 cm^3^ and that of the left adrenal gland was 9.46 ± 4.35 cm^3^; the difference was not statistically significant (p = 0.10, t = 1.65, df = 198). There was also no significant difference between right and left adrenal gland volumes in the control group (p = 0.44, t = 0.78, df = 38) (Table 1).

Relative adrenal gland weight was calculated by dividing the adrenal gland weight (in grams) by the square of the total height of the deceased (m^2^). The relative weight of the combined adrenal glands was 3.76 ± 1.01 g/m^2^, which was significantly higher in the study group than the control group (2.78 ± 0.53 g/m^2^; unpaired t-test, p = 0.0001, t = 4.21, df = 118). For the right adrenal gland, the difference between the study group (1.85 ± 0.05 g/m^2^) and the control group (1.42 ± 0.24 g/m^2^) was extremely significant (t-test, p = 0.0003, t = 3.75, df = 118). Relative weight for the left adrenal gland was 1.92 ± 0.54 g/m^2^ in the study group, which was significantly higher than the control group's 1.36 ± 0.30 g/m^2^ (p < 0.0001, t = 4.41, df = 118).

The external appearance of the adrenal gland was graded from 1 to 4, indicating pale, normal, congested, and hemorrhagic, respectively. Of the 200 adrenal glands examined from the 100 study cases (two adrenal glands from each individual), 60% of cases (n = 60 cases, 120 adrenal glands) had a hemorrhagic external appearance, graded as 4; 37% (n = 37 cases, 74 adrenal glands) were congested, with grade 3; 2% (n = 2 cases, 4 adrenal glands) had a pale appearance, graded 1; and 1% (n = 1 case, 2 adrenal glands) appeared normal and were graded as 2 (Table 1) (Figure 1). Among the 20 control cases and 40 adrenal glands examined, 8 cases (40%) and 14 adrenal glands (35%) were grade 1 (pale), 7 cases (35%) or 12 adrenal glands (30%) belonged to grade 2 (normal appearance), 8 cases (40%) or 14 adrenal glands (35%) were grade 3 (congested appearance), and none exhibited a hemorrhagic appearance or grade 4. In some cases, it was also observed that only one adrenal gland exhibited a change while the other did not (Table 1).

In the suicidal group, the combined weight of both adrenal glands (10.38 ± 2.74 g) was higher than in the control group (7.84 ± 1.63 g), and the difference in weight was extremely significant (unpaired t-test, p < 0.0001, t = 3.99, df = 118). In the suicidal group, the mean ± SD weight of the right adrenal gland was 5.098 ± 1.39 g; the left adrenal gland weighed more than the right adrenal gland (5.28 ± 1.45 g), but this difference in weight was not statistically significant (p = 0.366, t = 0.91, df = 198 by t-test). In the control group, the mean ± SD weights of the right and left adrenal glands were 3.99 ± 0.78 g and 3.84 ± 0.9 g, respectively; this difference was not significant (unpaired t-test, p = 0.55, t = 0.60, df = 38). A statistically significant difference was found in the weight of the right adrenal gland among the suicidal and control groups (unpaired t-test, p = 0.0009, t = 3.42, df = 118) and the weight of the left adrenal gland (p < 0.0001, t = 4.27, df = 118) compared to the control group (Table 1).

In the study group, the left adrenal gland weighed more than the right adrenal gland (5.43 ± 1.49 g vs. 5.38 ± 1.37 g, respectively), but the difference was not statistically significant. However, in the control group, the mean ± SD weight of the right adrenal gland (4.04 ± 0.80 g) was higher than that of the left (3.86 ± 0.93 g). Thus, it can be concluded that in suicidal individuals, the increase in total combined weight was due to an increase in the weight of the left adrenal gland. In males, the difference in weight of the right and left adrenal glands in the study and control groups was statistically very significant (t-test, p = 0.0003, t = 3.76, df = 83 for the right adrenal gland and p = 0.0001, t = 4.02, df = 83 for the left adrenal gland) (Table 2). In female suicidal individuals, the mean ± SD combined adrenal gland weight (9.01 ± 2.56 g) did not differ significantly from the control group's weight (7.35 ± 1.34 g) according to the unpaired t-test (p-value = 0.215, t = 1.26, df = 33), which contradicts the findings in men. The mean ± SD weights of the left adrenal gland in female suicidal subjects (4.55 ± 1.37 g) and in control subjects (3.74 ± 0.79 g) were higher than those of the right adrenal gland in suicidal (3.61 ± 0.54 g) and control (3.74 ± 0.79 g) females. The mean ± SD weight of the right adrenal gland in the female study group was not statistically different from that in the female control group (unpaired t-test, p = 0.19, t = 1.33, df = 33). The mean ± SD weight of the left adrenal gland in the female study group was also not significantly different from that of control females (p = 0.25, t = 1.14, df = 33) (Table 2).

**Table 2:** Gender-wise comparison of adrenal weight in the study and control groups N: Number of subjects *p < 0.05 was significant

Category	Male			Female		
	Right	Left	Combined	Right	Left	Combined
Study group (N=69 males, 31 females)	5.38±1.37	5.43±1.49	10.80±2.74	4.46±1.25	4.55±1.37	9.01±2.56
Control group (N=16 males, 4 females)	4.04±0.80	3.86±0.93	7.89±1.68	3.61±0.54	3.74±0.79	7.35±1.34
p-value	0.0003*	0.0001*	0.0001*	0.19	0.25	0.215

There was a significant difference in the weight of the combined adrenal gland between males and females in the suicidal group (t-test, p = 0.027, t = 3.08, df = 98). But in the control group, it was not significant (p = 0.56, t = 0.59, df = 18) (Table 2).

In the suicidal group, there was a significant relationship between the height of the individual and the weight of both adrenal glands (Pearson’s correlation coefficient for the right adrenal was r = 0.32, p = 0.001; for the left adrenal gland, r = 0.34, p = 0.002), but the relationship between adrenal weight and total body weight was not significant (Pearson’s correlation coefficient for the right adrenal was r = 0.174, p = 0.08, and r = 0.11, p = 0.26 for the left adrenal). The mean ± SD weight of the right adrenal was significantly correlated with the mean ± SD weight of the right kidney (Pearson’s correlation coefficient was r = 0.39, p = 0.001), and the weight of the left adrenal was also significantly correlated with the mean ± SD weight of left kidney (Pearson’s correlation coefficient was r = 0.28, p = 0.004) (Table 3).

**Table 3:** Relation of adrenal weight with total body weight and height of deceased and weight of kidney in study and control groups N: Number of subjects *p < 0.05 was significant

Study group						
Parameters (mean±SD)	Height of deceased (in meters) (1.66±0.08)		Weight of deceased (in kg) (56.94±8.39)		Weight of kidney (in gms) (right=113.3±11.3) (left=112.8±11.4)	
	Right	Left	Right	Left	Right	Left
Weight of adrenals (in gms)	5.01±1.5	5.28±1.4	5.01±1.4	5.28±1.4	5.01±1.4	5.28±1.4
p-value	0.001*	0.002*	0.08	0.26	0.001*	0.004*
Control group						
Parameters (mean±SD)	Height of deceased (in meters) (1.68±0.09)		Weight of deceased (in kg) (62.6±13.02)		Weight of kidney (in gms) (right=117.9±12.8) (left=117.7±12.7)	
	Right	Left	Right	Left	Right	Left
Weight of adrenals (in gms)	3.99±0.78	3.84±0.89	3.99±0.78	3.84±0.89	3.99±0.78	3.84±0.89
p-value	<0.05*	<0.05*	>0.05	>0.05	<0.05*	<0.05*

In control individuals, there was also a significant correlation between the total height of the deceased and the weight of both adrenal glands (Pearson’s correlation coefficient, r = 0.29, p<0.05 for the right adrenal; r = 0.32, p<0.05 for the left adrenal). The relationship between total body weight and the weight of both adrenal glands was not significant. (Pearson’s correlation coefficient was r = 0.21, p > 0.05 for the right and r = 0.18, p > 0.05 for the left adrenal gland) (Table 3).

Similar to the study group in the control group, there was also a significant relationship between the weight of the right kidney with the weight of the right adrenal gland and the left kidney with the left adrenal gland (Pearson's coefficient r = 0.34, p<0.05 for the right adrenal gland; r = 0.38, p<0.05 for the left adrenal gland) (Table 3).

## Discussion

Chronic stress alters the morphology of the adrenals; therefore, this study was carried out to examine the adrenals of individuals dying suddenly from causes other than suicide in order to obtain a standard of comparison for the adrenals of healthy subjects exposed to acute stress from dying and chronically stressed individuals. Many authors have proven that suicide results from a complex and multifaceted behavior pattern [7-10]. Careful post-mortem psychiatric investigations have revealed that most subjects who commit suicide are mentally ill [11-27]. In the present study, we were unable to confirm this view. We observed that 25%, 13%, 3%, and 8% had a history of chronic stress, self-destructive behavior, untreated depression, and a history of financial or marital stress, respectively. Since this psychiatric history was based on statements by relatives, investigating agencies, and bystanders, it was not complete or reliable enough to warrant a statement regarding the presence and duration of a specific psychiatric disease or stress before suicide. However, it can be hypothesized that 49% of the subjects had some sort of chronic stress before death. Assuming that none of the subjects had a severe, prolonged, or debilitating physical ailment at the time of death, we assumed that pre-existing psychiatric morbidity largely accounted for the observed adrenal gland changes in the suicidal group.

In the present study, the right adrenal gland was found to be heavier than the left in the control group. In addition, both adrenal glands weighed more in males. Researchers Quinan and Burger [28] and Dilruba et al. [29] found no evidence of a weight difference between the two glands. Nevertheless, Lam et al. [30], Singh et al. [31], Glass and Mundy [32], and Narongchai and Narongchai [33] noted that the left gland was heavier than the right. According to several studies, including those by Gelfman [34], Symington [35], Neville and O'Hare [36], Bagheri et al. [37], Rosai and Ackerman [38], and Stacy [39], the weight of the two adrenal glands is similar for men and women. However, Narongchai and Narongchai [33] found the gland to be heavier in women than in men. The reason for these different observations could be based on differences in the population groups or sizes included in studies by different authors.

Among the cases in the present study, the weight of the left adrenal gland was higher than the right, and the combined weight of the gland in males was higher than that in females. Dumser et al. [40] and Sarkar et al. [41] have reported similar findings, while Dorovini-Zis and Zis [42] found no weight difference, which could be due to the inclusion of a smaller number of subjects or a difference in the nature and duration of psychiatric morbidity preceding suicide. Dumser et al. [40] have also suggested that the left adrenal gland in the suicidal group was heavier because it was not squeezed between other organs and was liable to enlarge more easily in chronic stress conditions. Dorovini-Zis and Zis [42] found no gender-specific differences in weight. In the current study, the difference in weight among males was significant in both groups but was insignificant in females, probably due to their disproportionate ratio (31:4) in both groups. No significant relationship between adrenal gland weight and the age or weight of the individual was observed. Dorovini-Zis and Zis [42] made similar observations, showing that the increase in adrenal gland weight under chronic stress conditions was not dependent on the person's age or weight. However, Narongchai and Narongchai [33] also noted a significant correlation between the individual's height and adrenal gland weight in both groups; this observation suggests that in taller individuals, the weight of the adrenal gland is higher than in normal subjects.

According to the available literature, no work has previously assessed adrenal gland volume on autopsy in suicidal cases, as in the current study. Nemeroff et al. [43] studied adrenal gland volume in depressive patients and observed larger volumes among those patients as compared to normal controls. The results of the present study have shown that the difference in adrenal gland volume between the suicidal and control groups was not significant, indicating that the increase in size should not be taken as a differentiating criterion for acute versus chronic stress. Not all subjects who commit suicide meet the diagnostic criteria for major depression, and pathological activation of the hypothalamic-pituitary-adrenal axis has rather been primarily associated with the presence of depression and stress and not with suicide per se [44,45]. The size of the adrenal gland is closely associated with the functional activity of the gland, and previous researchers have proven that the change in the size of the adrenal gland is easily and rapidly reversible in a matter of days following a change in adrenal gland function [46-48]. It also suggests that the increased size of the adrenal gland is not an index of a cumulative lifetime of depression or chronic stress [44]. Therefore, while an enlarged adrenal gland isn't always a sign of a persistently stressed or fatigued adrenal gland, it does suggest that the adrenal gland is under some kind of stress, even if it isn't Cushing's syndrome or chronic stress.

To find the relative weight of the adrenal glands, we divided their mass by their surface area. This calculation was performed because the correlation of organ weight and body measures has seemed to be closer to that of the body surface area than to the body mass or the height alone. The relative adrenal gland weight was significantly higher in the suicidal group, similar to the observation of Dumser et al. [40]; however, the variation was only found in the numerical value. The reason for this effect may be most likely related to the method of determining the body surface area. Dumser et al. [40] determined this value from the nomogram derived from the equation of DuBois and DuBois (body surface area (m2) = weight (kg)^0.425^ × height (cm)^0.725^ × 0.007184), using the individual’s height and body weight; however, in the present study, the body surface area was simply taken as the square of the height in centimeters.

## Conclusions

The present study found that in the suicidal group, 60% of cases of adrenal glands had a hemorrhagic appearance and 37% had a congested appearance; however, in the control group, the hemorrhagic appearance of adrenal glands was not seen, although 40% were congested. Thus, the external appearance of the adrenal glands may be regarded as a normal response to stress in relation to the mode of death. Adrenal glands were heavier in males than in females. The left adrenal gland was more likely to show an increase in weight in response to chronic stress. The weight of the adrenal gland in both groups was significantly associated with the height of the deceased individual and the weight of the kidney, but not with the age or body weight of the deceased. Relatively higher adrenal gland weight can be considered specific for suicidal cases exposed to chronic stress. However, the volume of the adrenal gland may be considered to be an unreliable criterion for differentiating chronic stress from acute stress.
